# A Study of Visual and Musculoskeletal Health Disorders among Computer Professionals in NCR Delhi

**DOI:** 10.4103/0970-0218.58392

**Published:** 2009-10

**Authors:** Richa Talwar, Rohit Kapoor, Karan Puri, Kapil Bansal, Saudan Singh

**Affiliations:** Department of Community Medicine, VMMC & Safdarjung Hospital, Delhi, India

**Keywords:** Computer professional, ergonomics, health problems

## Abstract

**Objective:**

To study the prevalence of health disorders among computer professionals and its association with working environment conditions.

**Study design:**

Cross sectional.

**Materials and Methods:**

A sample size of 200 computer professionals, from Delhi and NCR which included software developers, call centre workers, and data entry workers.

**Result:**

The prevalence of visual problems in the study group was 76% (152/200), and musculoskeletal problems were reported by 76.5% (153/200). It was found that there was a gradual increase in visual complaints as the number of hours spent for working on computers daily increased and the same relation was found to be true for musculoskeletal problems as well. Visual problems were less in persons using antiglare screen, and those with adequate lighting in the room. Musculoskeletal problems were found to be significantly lesser among those using cushioned chairs and soft keypad.

**Conclusion:**

A significant proportion of the computer professionals were found to be having health problems and this denotes that the occupational health of the people working in the computer field needs to be emphasized as a field of concern in occupational health.

## Introduction

In the twenty-first century, computers have become almost as ubiquitous as the humble pen and paper in many peoples' daily life. There are approximately six computers per thousand population with an installation of 18 million personal computers (PCs) and their number is increasing all the time.(1) The computer is a vital tool in every dimension. However, the long periods of working at a computer as most people do, can cause musculoskeletal problems, eyestrain, and overuse injuries of the hands and wrists which can be reduced or eliminated with proper workstation design and improved posture. A survey done by the American Optometric Association estimates that at least 10 million cases of computer-related eyestrain were reported each year.(2) The proliferation of video display terminals (VDT), in the modern office setting has generated concern related to potential health hazards associated with their use.(3) Using the wrong chair or just sitting improperly in front of a computer for long time can lead to chronic debilities such as stiffness, headache, and backache. Muscles and tendons can become inflamed due to greater periods of sitting on PC's. Carpal tunnel syndrome is a common example of an overuse injury associated with computer work. This painful disorder of the hand is caused by pressure on the main nerve that runs through the wrist. The fingers are also prone to overuse injury, particularly the finger that clicks the mouse buttons.(2)

The human eye basically prefers to look at the objects greater than 6 m away, thus work done on computer demands a close-up view which strains eye muscles and thereby leads to eye fatigue. Surveys of computer workers reveal that vision-related problems are the most frequently reported health- related problems, occurring in over 70% of computer workers.(5) Computer vision syndrome is related to the unique aspects of the task. Working at a computer is more visually demanding than doing other standard office work such as reading printed documents. Aspects of the design of the computer video display such as screen resolution and contrast, image refresh rates and flicker, and screen glare, as well as working distances and angles all may contribute to worker symptoms.(6,7) This study was done to determine the prevalence of visual and musculoskeletal health disorders and the associated factors among computer professionals in NCR Delhi.

## Materials and Methods

The present study was a cross-sectional analysis done among computer professionals working in Delhi, in June 2007. A total of 200 computer professionals were purposively selected for the study. One computer-based organization's office was selected from four parts of Delhi – North, East, South and West, and one from the National Capital Region (NCR) Gurgaon. A minimum of 30 professionals was sampled from each selected center. Inclusion criteria were as follows: the respondents included people who had been working in the current job for at least six months, and who work on the computer for at least 3 h per day continuously. Only those subjects were taken who were present at the time of our visit. Computer professionals included software developers, call centre workers, and data entry workers.

Permission for conducting the study was obtained from the various offices prior to the initiation of the study. The study subjects were explained the purpose of study and were assured about the confidentiality and anonymity of the information so obtained. Data was collected by a self administered pre-structured, pre-tested questionnaire that included details such as age, sex, working hours, details of the working environment, and detailed information about experiencing of various visual or musculoskeletal problems. The data thus collected was converted into a computer-based spreadsheet and analyzed by studying the proportions and associations by applying Chi-square test.

## Results

The present study included 200 computer professionals viz. software developers (78), call center workers (56), and data entry workers (66). Mean age of study subjects was 28.23 with 58.5% of subjects being in the age group of 20-29 years.The male: female ratio was 5:3 among the respondents. Regarding hours at work spent in front of a computer, 60 (30%) reported spending 3-6 h/day, 88 (44%) spent 6-9 h, while 52 (26%) of the respondents spent more than 9 h/day working on a computer.

The prevalence of visual problems found in the study group was 76% (152/200), and musculoskeletal problems were reported by 76.5% (153/200). The subjects were asked about the various visual problems experienced by them. The results are shown in Table 1. Among the visual problems, most common were watering of eyes (23.2%), pain in eyes (25.7%), irritation in eye (18.6%), burning/itching (29.8%), redness (40.7%), blurring of vision (13.2%), and headache (29.2%). Table 2 depicts that the common musculoskeletal problems were: pain/stiffness in neck (48.6%), pain/stiffness in shoulder (15.7%), pain/stiffness in lower back (35.6%), and pain/stiffness in wrist, hand, or fingers (23.1%).

**Table 1:** Distribution of visual problems among the study subjects reporting them (*N* = 152)

Visual problem	Percentage (n)	%age Males	%age Females
Watering of eyes	23.2 (35)	14.5	8.7
Pain in eye	25.7 (39)	16.0	9.6
Irritation in eye	18.6 (28)	11.6	6.9
Burning/itching	29.8 (45)	18.6	11.1
Redness of eye	40.7 (61)	25.4	15.2
Blurring of vision	13.2 (21)	8.8	4.4
Headache	29.2 (44)	17.1	12.0

*Responses are not mutually exclusive

**Table 2:** Distribution of musculoskeletal problems in study subjects reporting any problems (*N* = 153)

Musculoskeletal problem	Percentage (n)	%age Males	%age Females
Pain/stiffness in neck	48.6 (73)	34.7	13.8
Pain/stiffness in shoulder	15.7 (23)	10.4	5.2
Pain/stiffness in lower back	35.6 (54)	25.4	10.1
Pain/stiffness in wrist/hand/fingers	23.1 (36)	16.5	6.6

*Responses are not mutually exclusive

The association of visual and musculoskeletal problems with the time being spent on computer per day was analyzed. The results are depicted in Table 3. It was found that there was a gradual increase in visual complaints as the number of hours spent working on computers daily increased and the same relation was found to be true for musculoskeletal problems as well.

**Table 3:** Distribution of computer related health problems by average time spent daily working on computer (in hours/ day)

Computer hours/day	Visual	Musculoskeletal
	N (%)	N (%)
3-6h	36 (61.0)	42 (71.1)
6-9h	72 (80.9)	69 (77.5)
9h or more	44 (84.6)	42 (80.7)
Total (having the problem)	152 (76.0)	153 (76.5)
*P* value	0.005	0.47

The association of visual and musculoskeletal problems with the ergonomic condition of the respondent's working environment was analyzed. Antiglare screen was found to be protective against visual problems. Visual problems were reported by only 53.4% (31/58) among those using antiglare screen, compared to 85.2% (121/142) among those who were not using antiglare screen (χ^2^ = 22.78, *df*=1, *P* = <0.001). Visual problems were reported by only 51.7% (30/58) among those working in adequate lighting, compared to 85.9% (122/142) among those working in inadequate lighting conditions (χ^2^ = 26.93, *df* = 1, *P* = <0.001).

The association of musculoskeletal problems with the ergonomic conditions was also studied. Musculoskeletal problems were reported by only 48.7% (38/78) among those using cushioned chairs, compared to 94.2% (115/122) among those who were not using cushioned chairs (χ^2^ = 54.89, *df* = 1, *P* = <0.001). Musculoskeletal problems were reported by 70% (70/100) among those using soft keypad, compared to 83 % (83/100) among those not using soft keypad (χ^2^ = 4.7, *df* = 1, *P* = 0.03).

## Discussion

We studied visual and musculoskeletal problems among professionals working in computer-related fields in national capital region (NCR) Delhi. Nearly three-fourths of the respondents had visual problems as well as musculoskeletal problems. Magnitude of visual problems was found to be directly related to average computer hours per day. Visual problems were less in persons using antiglare screen, and those with adequate lighting in the room. This could mean that putting emphasis on ergonomic conditions under which computer-related work is being performed, can go a long way in reducing the burden of visual problems. Musculoskeletal problems were greater in professionals working for a longer time per day, and were found to be significantly lesser among those using cushioned chairs and soft keypad.

The study results for visual health problems are consistent with the study conducted by Sharma *et al*.(1) Headache encountered in our study was 29.2% which is concordant with an earlier study by Bhatt.(4) The results regarding musculoskeletal problems were also largely similar to those found by Sharma *et al*. The prevalence of various computer related problems is not only dependent on the type of profession but also on the environment of the working place and posture adopted. Sources of information such as internet, seminars, and lectures can help in raising the awareness rates regarding computer- related problems.(1) Limitation of our study was that it was a purposive sampling and we took only day-shift workers.

In conclusion, nearly three fourths of the computer professionals we studied had mentioned to have some computer-related health problems. This is a significant proportion and denotes that the occupational health of the people working in the computer field should be emphasized as a field of concern in public health. The ergonomics of the working environment of the computer professionals have a direct impact on their well being. Hence the organizations employing them, as well as the professionals themselves need to be sensitized regarding the importance of the regular health checkups and proper working conditions.
